# Loneliness, Marital Quality, and Vascular Health Among Older U.S. Couples: A Longitudinal Dyadic Study

**DOI:** 10.1093/geroni/igaa057.2041

**Published:** 2020-12-16

**Authors:** Jeffrey Stokes, Adrita Barooah

**Affiliations:** University of Massachusetts Boston, Boston, Massachusetts, United States

## Abstract

Loneliness is a contributor to later life declines in health, including vascular health. Importantly, loneliness is not restricted to those who lack close social ties: More than one-third of married U.S. older adults experience loneliness, and having a lonely spouse increases the likelihood of experiencing loneliness oneself. Thus, over time loneliness in either spouse may lead to worse health for both spouses. Using longitudinal dyadic data from the Health and Retirement Study (2008-2014), we estimated multilevel lagged dependent variable models to examine implications of both partners’ loneliness at baseline for each spouse’s HbA1c four years later. Findings revealed that effects of both partners’ loneliness were contingent upon marital quality: Own and partner’s loneliness led to increases in HbA1c when perceived marital support was low, but this was attenuated at higher levels of marital support. These results extend prior research concerning loneliness and vascular health, and loneliness as a relational experience.

